# Effective inactivation of Nipah virus in serum samples for safe processing in low-containment laboratories

**DOI:** 10.1186/s12985-020-01425-8

**Published:** 2020-10-09

**Authors:** Shumpei Watanabe, Shuetsu Fukushi, Toshihiko Harada, Masayuki Shimojima, Tomoki Yoshikawa, Takeshi Kurosu, Yoshihiro Kaku, Shigeru Morikawa, Masayuki Saijo

**Affiliations:** 1 Department of Microbiology, Faculty of Veterinary Medicine, Okayama University of Science, 1-3 Ikoinooka, Imabari, Ehime 794-8555 Japan; 2 Department of Virology I, National Institute of Infectious Diseases, 4-7-1 Gakuen, Musashimurayama, Tokyo 208-0011 Japan; 3 Management Department of Biosafety and Laboratory Animal, National Institute of Infectious Diseases, Tokyo, Japan; 4 Division of Veterinary Science, National Institute of Infectious Diseases, Tokyo, Japan; 5 Department of Virology I, National Institute of Infectious Diseases, 1-23-1 Toyama, Shinjuku, Tokyo 162-8640 Japan

**Keywords:** Nipah virus, Virus inactivation, Diagnosis, Biosafety level 4, Virus stability

## Abstract

**Background:**

Nipah virus (NiV) is an emerging zoonotic paramyxovirus that causes severe encephalitis and respiratory disease with a high mortality rate in humans. During large outbreaks of the viral disease, serological testing of serum samples could be a useful diagnostic tool, which could provide information on not only the diagnosis of NiV disease but also the history of an individual with previous exposure to the virus, thereby supporting disease control. Therefore, an efficient method for the inactivation of NiV in serum samples is required for serological diagnosis.

**Methods:**

We determined the optimal conditions for the inactivation of NiV infectivity in human serum using heating and UV treatment. The inactivation method comprised UV irradiation with a cover of aluminum foil for 30 min and heating at 56 °C for 30 min.

**Results:**

With an optimized protocol for virus inactivation, NiV infectivity in serum samples (containing 6.0 × 10^5^ TCID_50_) was completely inactivated.

**Conclusions:**

We developed a recommended protocol for the effective inactivation of NiV. This protocol would enable a regional or local laboratory to safely transport or process samples, including NiV, for serological testing in its biosafety level-2 facility.

## Background

Nipah virus (NiV), a member of the genus *Henipavirus* belonging to the family *Paramyxoviridae*, is an enveloped virus with a non-segmented, negative-strand RNA genome [1]. NiV was first discovered in 1998–99 as the etiological agent responsible for an outbreak of a severe respiratory disease in pigs, as well as encephalitis and respiratory disease in humans (276 recorded cases, 40% mortality) in Malaysia and Singapore [1–3]. Subsequent outbreaks of NiV disease have been reported annually in Bangladesh and India. The infection of humans with the Bangladeshi or Indian NiV strains resulted in a higher percentage of patients with respiratory disease and a higher mortality rate (70%), compared to that with Malaysia and Singapore strains [2, 3]. Owing to its high lethality, NiV is generally classified as a biosafety level-4 (BSL-4) pathogen.

Fruit bats of the genus *Pteropus* are the natural reservoirs of NiV [3, 4]. Based on molecular or serological surveys in these bats, NiV is distributed widely in Southeast Asia [3, 5–8]. In addition to the multiple species of bats, various mammalian species, including pigs, horses, and humans, can be accidentally infected with NiV [9]. Furthermore, person-to-person transmission has been recorded in some cases of NiV disease outbreaks [10, 11]. Based on these features, the World Health Organization has listed NiV disease as a priority disease in the blueprint of research and development [12].

In cases where an outbreak of NiV disease occurs in a NiV-free country, virological tests would be required for diagnosis. Clinical samples such as blood and other body fluids should be manipulated in high-containment (BSL-4 or BSL-3) laboratories, making it difficult to perform virological tests in laboratories without high-containment facilities. To overcome this difficulty, an effective inactivation process for handling clinical samples without causing degradation of the samples is necessary. Proper inactivation of the samples makes it possible to manipulate them in BSL-2 laboratories for virological tests. Thus, it is desirable to develop a protocol for the effective inactivation of NiV in clinical samples for safe transportation and processing in BSL-2 laboratories.

As part of the standard settings, BSL-2 laboratories are generally equipped with heating and UV irradiation equipment. Thus, the objective of this study was to explore optimal conditions for the inactivation of NiV in serum samples subjected to heating and UV treatment. Methods for the inactivation of infectious NiV in serum samples, which should be used for serological analyses, have not yet been established. Serum samples from the patients potentially contain infectious NiVs, since it is thought that the virus causes multi-organ failure via viremia [9, 13].

Although the stability of NiV in some liquids (growth media, urine, and juice) at environmental temperature has been reported [14], the stability of infectious NiV in the sera has not yet been characterized. Thus, the stability of NiV in serum samples at room temperature was also examined.

## Materials and methods

### Cell lines and viruses

Vero cells were maintained in Dulbecco's modified Eagle’s medium (DMEM; Sigma, St. Louis, MO) supplemented with 10% fetal bovine serum (FBS, DMEM-10FBS). The NiV strain, Ma-JMR-01-98, was kindly provided by Dr. Kouichi Morita (Institute for Tropical Medicine, Nagasaki University, Nagasaki, Japan). The virus was propagated in Vero cells. At 2 days post-infection, the infectious fluid was harvested. Cellular debris was removed by low-speed centrifugation (2000 × *g*, 10 min, 4 °C) and the supernatant was collected. Experiments using infectious NiV were conducted in BSL-3 or BSL-4 laboratories under the biosafety regulation of the National Institute of Infectious Diseases, Tokyo, Japan.

### Heat treatment and UV irradiation

**Fig. 1:**
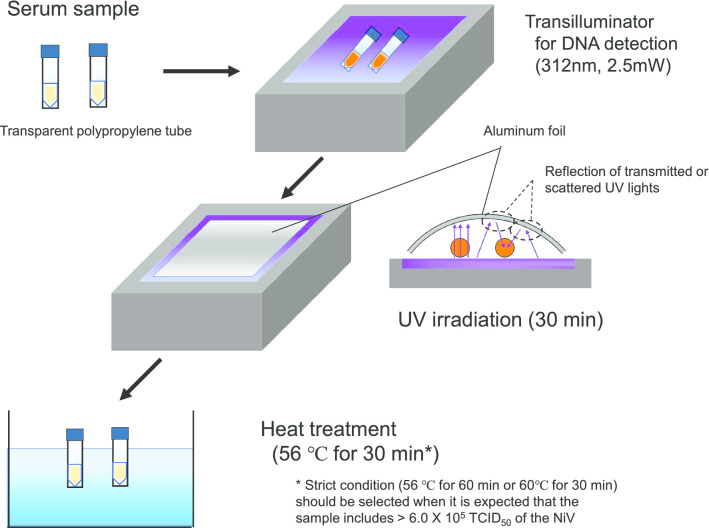
Schematic diagram of a protocol for effective inactivation of NiV in serum samples. Two hundred microliter of serum samples should be used for the protocol as start materials, since efficacy of the shown protocol was confirmed with the volume

The NiV strain Ma-JMR-01-98 (3.0 × 10^7^ 50% tissue culture infectious dose (TCID_50_)/mL) was diluted tenfold in DMEM-10FBS or in pooled human serum (Tennessee Blood Services, Memphis, TN) to prepare virus samples (3.0 × 10^6^ TCID_50_/mL) for the inactivation experiments. Next, 200 µL of the virus sample was aliquoted into a 1.5 mL transparent polypropylene tube (No.72.692S, Sarstedt, Nümbrecht, Germany). The virus samples were then processed by heating at 56 °C or 60 °C for 30 or 60 min or by UV irradiation (wavelength, 312 nm; output power, 2.5 mW/cm^2^) for 10 min or 30 min using a transilluminator (model TPP-10M, Vilber-Lourmat, Collégien, France). The distance between the UV outlet and samples was approximately 1.0 cm and the UV intensity was 860 μW/cm^2^. To ensure the delivery of UV irradiation onto the whole surface of the sample tubes, tubes on the transilluminator were covered with aluminum foil, which reflects UV effectively [15], as shown in the schematic overview of the inactivation method (Fig. 1). All samples were stored at − 80 °C until virus titration, and the samples after single freeze–thaw cycle were used for the titration.

### Virus titration

The infectious dose of NiV in the samples with or without heating and/or UV-treated samples was determined as described previously [14]. Briefly, all samples were serially tenfold diluted with DMEM, and 33 μL volumes of each dilution were placed onto the confluent monolayers of Vero cells cultured in 96-well microplates. Following a 3-day incubation at 37 °C in 5% CO_2,_ the plates were scored for cytopathic effect (CPE), and the TCID_50_ was determined.

### Confirmation of complete inactivation of spiked NiV by virus isolation

Two hundred microliters of DMEM-10FBS or pooled human serum spiked with infectious NiV (6.0 × 10^5^ TCID_50_) was prepared as described above. The sample-containing tubes with aluminum foil lids were then treated with UV irradiation for 30 min. Subsequently, the UV-treated tubes were further heated at 56 °C for 30 min in a water bath. The samples with the spiked virus, which were not subjected to the treatment, were used as controls. Triplicate samples with the treatment and the controls were tested for virus isolation using Vero cells. Vero cells seeded in a 6-well plate were inoculated with 100 μL of each sample. CPE in the cells was monitored for 6 days, following which the plates were stored at − 80 °C. After thawing the plates, the supernatant of the culture fluid was collected by low-speed centrifugation (2000 × *g*, 5 min), and 900 μL of the supernatant was transferred onto Vero cells cultured in a new plate. After 1 h incubation, the supernatant was replaced with fresh growth medium (DMEM-10FBS), and the cells were incubated for 6 or 7 days to observe the CPEs. This blind passage was repeated three times.

### Stability of NiV spiked in pooled serum at room temperature

The stock of NiV containing 3.0 × 10^7^ TCID_50_/mL was diluted tenfold in the pooled human serum to prepare the virus samples (3.0 × 10^6^ TCID_50_/mL). Two hundred microliters of the virus sample in the transparent polypropylene tube (No.72.692S, Sarstedt) was then incubated at 25 °C for 7 days, followed by storage at − 80 °C. In contrast, the virus sample was desiccated by spreading 20 µL onto 6-well plastic plates and air drying at 25 °C within 7 days. The virus was recovered at intervals by resuspension in 200 µL of DMEM-10FBS and stored at − 80 °C. The infectious doses of all the samples stored were determined in Vero cells as described above.

## Results

### Thermal stability of NiV

**Fig. 2:**
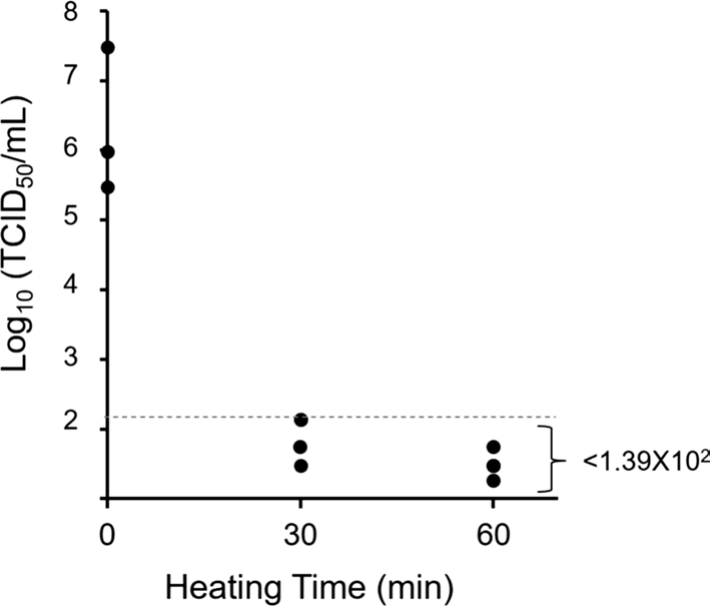
Heat inactivation of NiV in 90% human serum. Aliquots (200 μL) of NiV (3.0 × 10^6^ TCID_50_/mL) were treated at 56 °C for the indicated times. NiV remaining in the samples were tested for the determination of infectious titers in Vero cells. The virus titers (TCID_50_/mL) in the treated samples were determined as described in the Materials and Methods section. Heat treatments were performed in triplicate. The limit of detection (1.39 × 10^2^ TCID_50_/mL) is shown as a dashed line

Because certain RNA viruses are not necessarily inactivated by heating at 56 °C for 30 min, the inactivation efficacy of the heating and UV-radiation conditions (56 °C for 30 or 60 min and 60 °C for 30 min) was tested to evaluate the thermal stability of NiV. The viral titers of NiV spiked in pooled human serum before and after the heat treatment were determined (Fig. 2). After heating at 56 °C for 30 min, the NiV titer (1.04 × 10^7^ TCID_50_/mL) decreased by more than 4 log_10_. In the triplicate test, the titer decreased to the detection limit (1.39 × 10^2^ TCID_50_/mL) twice, whereas the titer decreased below the detection limit once (< 1.39 × 10^2^ TCID_50_/mL) (Fig. 2). The treatments at 56 °C for 60 min and with 60 °C for 30 min resulted in NiV infectious titers below the detection limit in all triplicate tests (Fig. 2 and data not shown).

### Efficacy of UV irradiation on NiV inactivation

**Fig. 3:**
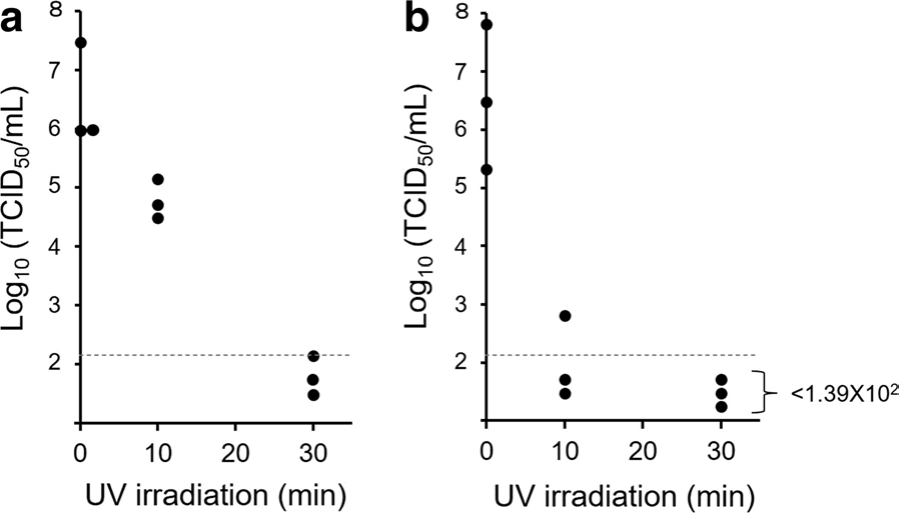
Inactivation of NiV using UV irradiation. Aliquots (200 μL) of NiV (3.0 × 10^6^ TCID_50_/mL) in 90% human serum (**a**) or growth medium (DMEM-10FBS) (**b**) were exposed to UV irradiation for the indicated times. The virus titers (TCID_50_/mL) in the treated samples are shown. UV treatments were performed in triplicate. The limit of detection (1.39 × 10^2^ TCID_50_/mL) is shown as a dashed line

To examine whether NiV is inactivated by UV irradiation (Fig. 3), transparent clear tubes containing infectious NiV solutions were exposed to UV irradiation for 10 and 30 min. NiV spiked into DMEM-10FBS decreased to the infectious dose level by the 10-min-UV irradiation with titers of < 1.39 × 10^2^ TCID_50_/mL, < 1.39 × 10^2^ TCID_50_/mL, and 6.47 × 10^2^ TCID_50_/mL in the triplicate test (Fig. 3b). In contrast, the infectious NiV spiked in pooled human sera was not efficiently inactivated by the 10-min-UV irradiation and was reduced by less than 2 log_10_ (Fig. 3a). This suggests that excess serum protein in serum samples interferes with the inactivation effect of UV-irradiation. However, even with 90% human serum, 30-min-UV irradiation decreased the infectious NiV titers almost completely, with the titer falling below the detection limit twice in the triplicate test (Fig. 3a).

### Confirmation of spiked NiV inactivation

Since the tested conditions for heating or UV irradiation reduced the virus titers to the level of the detection limit, we combined both methods to enhance the efficacy of the virus inactivation (Fig. 1). After the infectious NiVs (6.0 × 10^5^ TCID_50_) in human serum or DMEM-10FBS were processed with the inactivation protocol we designed, the treated samples were subjected to virus isolation in Vero cells. Infectious NiV was not isolated during the three cycles of blind passages in any of the treated samples but was isolated from the controls (Table 1).

**Table 1:** Virus isolation from the inactivated NiV using a protocol shown in Fig. 1

	No treatment		UV (30 min) and heating (56 °C for 30 min) in combination	
	In 90% human serum	In GM	In 90% serum	In GM
7 dpi	+	+	−	−
In blind passages	NT	NT	−	−

+ CPEs positive

− CPEs negative

*GM* Growth medium (DMEM-10FBS), *dpi* days post-infection, *NT* not tested

### Stability of NiV at room condition in a low-containment laboratory

**Fig. 4:**
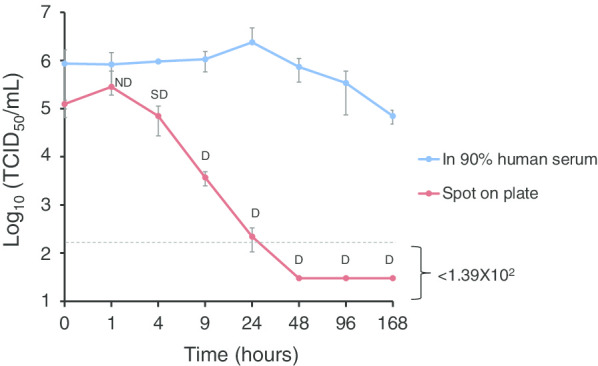
NiV survival after desiccation in 90% human serum. Two hundred microliter- aliquots of NiV in pooled human serum, containing 6.0 × 10^5^ TCID_50_, were incubated at 25 °C for the indicated times. In contrast, 20 µL of the above aliquot was spotted onto a plastic plate and air-dried at 25 °C for the indicated times. Immediately, viruses were recovered by resuspension in 200 µL of growth media at each time point. The virus titers (TCID_50_/mL) in the treated samples were determined. The averages and standard deviations of three experiments are shown. The limit of detection (1.39 × 10^2^ TCID_50_/mL) is shown as a dashed line. Visual feature of the air-dried spots on the plate is shown as D; completely dried (only a smear), *SD*; semi-dried, or *ND* Not dried

The stability of NiV was examined, especially in pooled human sera, including large numbers of serum proteins, at 25 °C. The infectivity of NiV spiked in pooled sera in the liquid phase was tested for infectivity after incubation at 25 °C at the designated time points (Fig. 4). The infectivity in the solid phase was also tested by spotting a small portion of the serum, including the virus (20 µL), onto a plastic plate. After 4 h of incubation at 25 °C, the serum droplet was almost fully dried (semi-dry) on the plate, while the droplet was completely dried at 9 h (Fig. 4). The infectivity of the spiked virus decreased below the detection limit level at 24 h. The titers on the spots were 3.0 × 10^2^ TCID_50_/mL, 1.39 × 10^2^ TCID_50_/mL, and < 1.39 × 10^2^ TCID_50_/mL in the triplicate tests (Fig. 4). Once the spot was dried fully, NiV was almost inactivated within 15–20 h. In contrast, the titer of the spiked virus in pooled serum remained high even after 1 week with only 1.08 log_10_ reduction (Fig. 4).

## Discussion

Viral RNA (vRNA) detection based on RT-PCR techniques using various clinical samples or antibody detection using serum samples from patients in the acute and convalescent phases of diseases is commonly used for diagnosis. For vRNA detection, the methods for the complete inactivation of clinical samples are relatively established. For instance, samples containing RNA viruses or virus-infected cells are mixed with the commercially available guanidinium isothiocyanate buffers such as AVL and RLT buffers of the RNA extraction kits provided by QIAGEN (Valencia, CA), and then ethanol is added for complete chemical inactivation [16–18]. Conversely, protocols for the chemical inactivation of serum samples used for serological tests are not determined, since the chemical reagents interfere with the quality in serological tests. Typically, serum samples are treated first by heat inactivation at 56 °C for 30 min to minimize the effects of complement on the results of antibody-based assays, and this heating step was thought to eliminate the infectious virus. Although the heating of serum at 56 °C for 30 min is enough to eliminate the infectivity of dengue virus [19], previous studies have demonstrated that a longer incubation time (e.g., 60 min) at 56 °C, or incubation at a higher temperature, is required for complete inactivation of many RNA viruses because of their various thermal stabilities [20–24]. In the current study, we demonstrated that the conventional procedure for inactivating complement in serum (56 °C for 30 min) almost eliminated the infectivity of NiV in pooled human serum. This suggests that the conventional procedure of heating significantly lowers the risk of acquiring accidental infection through manipulation of serum samples, potentially including infectious NiV. However, it was notable that we detected a residual of the live virus once with the titer corresponding to the limit of detection after the heat treatment (Fig. 2), whereas no infectious NiV was detected under strict conditions (56 °C for 60 min and 60 °C for 30 min) in the triplicate test (Fig. 2 and data not shown). This might indicate that the latter conditions were required to ensure complete inactivation of the serum samples. It might be a rare case that the serum samples of patients contained more than 1 × 10^7^ TCID_50_/mL of NiV, compared with the levels of viral load reported in an animal model of nonhuman primate [25, 26]. Even with the strict conditions for heat inactivation, a single step of heat treatment would not guarantee complete inactivation. Recently, we reported a similar examination for the effective inactivation of a virus, severe fever with thrombocytopenia syndrome virus (SFTSV) [27]. In the report, CPEs were accidentally observed in some trials of virus isolation from clinical serum samples after heating to 60 °C for 60 min, although we had already demonstrated that the same condition fully eliminated the infectivity of SFTSV in the triplicate test of condition check. This inconsistency may be attributed to the difficulty in heating sample tubes evenly with conventional heating devices (water bath or heat blocker), as water often condenses on the lid of the tubes during heating. Thus, we think that the combination of another method with heat treatment would ensure complete inactivation compared with seeking the best condition for the single-step treatment of heating.

In addition to heat inactivation, UV and gamma irradiation can be frequently used for virus inactivation [27–29]. Special apparatuses are required for effective inactivation using UV and gamma irradiation, and these devices are not necessarily equipped in low-containment laboratories or regional bases for testing. Transilluminators for DNA detection (with a long wavelength, 312 or 365 nm) are normally found in regional laboratories, although a specific transilluminator with a shorter wavelength (e.g., 254 nm) or a UV crosslinker with adjustable related parameters is required to destroy viral genomes effectively by UV irradiation. Therefore, in this study, a transilluminator with a wavelength of 312 nm was employed to examine the suitable conditions for the effective inactivation of NiV in low-containment laboratories (Fig. 1). Moreover, to maximize the delivery of UV irradiation into serum samples, a clear polypropylene tube with high transparency for UV light was selected as a container for serum samples. Furthermore, sample tubes placed on the transilluminator were all covered with aluminum foil (Fig. 1) to reflect the transmitted and scattered UV lights to the target sample [15]. Whereas UV irradiation for 10 min effectively inactivated the infectious NiV spiked in the growth medium (Fig. 3b), the virus in pooled human serum was not eliminated by the 10-min irradiation (more than 1 × 10^4^ TCID_50_/mL of NiV remained inactivated) (Fig. 3a). This result suggests that excess serum proteins prevent the virus from being UV-irradiated. However, 30-min UV irradiation of the pooled human serum spiked with infectious NiV was effective for virus inactivation (Fig. 3a). Although we demonstrated that UV irradiation is an easy and effective method for the inactivation of NiV, we should consider that each viral genome of the mixture partially damaged by UV light could compensate for their damage to each other via RNA recombination. In paramyxovirus, events of RNA recombination have been reported in natural infection [30, 31]. Thus, the combination of other methods with UV inactivation would ensure the efficacy of inactivation.

Based on the sensitivities of NiV to heating and UV irradiation, we designed a recommended protocol for the effective inactivation of the virus in serum samples, which can be applied for the safe processing of samples in low-containment laboratories (Fig. 1). Using this protocol, we succeeded in inactivating 6.0 × 10^5^ TCID_50_ of NiV spiked in pooled human serum. In the triplicate test for virus isolation and subsequent blind passages, no CPEs were observed. Moreover, it was shown that the protocol combining heating and UV irradiation could completely eliminate virus infectivity (Table 1). In the confirmation of full inactivation, we performed UV- irradiation step at first before heating, to exclude the possibility that the residual water on the surface of tubes after heating with water bath could affect efficacy of the UV-radiation. But, for treatment of clinical serum samples, heating step could be performed before UV- irradiation. On the other hand, if the virus load in the serum of patients is thought to exceed 1 × 10^7^ TCID_50_ /mL, the heating condition could be replaced with the stricter condition (60 °C for 30–60 min). Importantly, heating serum samples even at 60 °C for 30 min never affected or interfered with ELISA titers against SFTSV in our prior report, whereas heating at 60 °C for 60 min slightly decreased their titers [27]. Moreover the 10-min UV treatment using the same transilluminator employed in the current study, with which infectious SFTSVs were completely inactivated in tested serum samples, did not at all decrease the ELISA antibody titer.

Additionally, we quantified the virus RNAs in the serum sample with spiked NiV containing 6.0 × 10^5^ TCID_50_ (200uL, 90% human serum), by SYBR real-time RT-qPCR using the reported N primer set (142 bp-long target) [32]. Then, the average copy number of the triplicate samples without the inactivation was approximately 8.5 × 10^5^, whereas that of the triplicate samples after treatment with the recommendation protocol was approximately 9.6 × 10^4^ as shown in Additional File 1 (Additional file 1: Fig. 1, and Additional file 1: Materials and Methods). This may indicate that the serum sample after the inactivation method is still available for vRNA detection, with decrease in sensitivity.

While the protocol for the effective inactivation of NiV was determined in the study, we need to evaluate the risks of processing serum samples in clinical settings, especially in the space outside the bio-safety cabinet. Regarding NiV, it is known that the virus is relatively stable in the liquid phase (growth media, urine, and fruit juice) at the environmental temperature [14]. Thus, NiV in serum samples with a large number of serum proteins is expected to be stable. In this study, NiV spiked in pooled human serum survived for a long time (Fig. 4). The infectious titer was only reduced by 1.08 log_10_ after leaving it to stand for 1 week at room temperature in the liquid phase. In contrast, the infectivity of NiV at the spot of the virus solution completely dried in the solid phase and quickly decreased within 15–20 h. This might mean that keeping the space for processing samples dried continuously decreases the risk of infection of NiV in laboratories used for serological tests.

## Conclusions

We determined the optimal conditions for thermal heating and UV irradiation for the inactivation of NiV in serum samples and growth medium and established a recommended protocol for NiV inactivation applicable in low-containment laboratories. Effective inactivation of NiV with the protocol would enable regional or local laboratories to manipulate clinical samples for serological assays such as neutralization tests as well as serological tests without infectious NiV, which we had developed before [33–35]. This will help in the rapid diagnosis of NiV disease in BSL-2 laboratories or regional bases for testing and in controlling large epidemics of the disease in future.

## Supplementary information

**Additional file 1**: **Figure 1**. Quantification of NiV RNAs of the serum sample with spiked NiVs containing 6.0 ×10^5^ TCID_50_ (90% human serum) before or after the inactivation treatment by the protocol shown in Fig. 1. The data indicate means ± standard deviations (SD).

## Data Availability

The datasets used and/or analyzed in the current study are available from the corresponding author upon reasonable request.

## Abbreviations

NiV: Nipah virus
BSL-4: Biosafety level-4
DMEM: Dulbecco's modified Eagle’s medium
FBS: Fetal bovine serum
DMEM-10FBS: DMEM supplemented with 10% FBS
NIID: National Institute of Infectious Diseases
TCID_50_: 50% Tissue culture infectious dose
CPE: Cytopathic effect
vRNA: Viral RNA
SFTSV: Severe fever with thrombocytopenia syndrome virus
