# Supplementary material for: Effective inactivation of Nipah virus in serum samples for safe processing in low-containment laboratories
Source: Virol J. 2020 Oct 9;17:151. doi: 10.1186/s12985-020-01425-8 (PMC7547523; doi:10.1186/s12985-020-01425-8)
Supplement: Supplementary file 1 — Additional file 1: Figure 1. Quantification of NiV RNAs of the serum sample with spiked NiVs containing 6.0 ×10^5^ TCID50 (90% human serum) before or after the inactivation treatment by the protocol shown in Fig. 1. The data indicate means ± standard deviations (SD). [file 12985_2020_1425_MOESM1_ESM.doc]

**Additional File 1**

Watanabe et al.

**Supplementary Materials and Methods**

Reverse transcription-quantitative PCR (RT-qPCR)

RT-qPCR was performed based on the prior report by Feldman et al (1). Briefly, total RNAs were extracted from the serum samples with spiked NiVs containing 6.0 ×10^5^ TCID_50_ (200uL, 90% human serum) with or without the treatment of inactivation (UV irradiation for 30min following heating at 56°C for 30 min) using High Pure Yield Viral RNA kit (Roche, Mannheim, Germany). The extracted RNAs were treated with RQ1 RNase-Free-DNase (Promega, Maddison, WI, USA), and reverse transcribed into cDNAs using SuperScript III reverse transcriptase (Invitrogen, Carlsbad, CA, USA) with Oligo dT primer. The cDNAs of the viral RNA genome was quantified using SYBR Premix Ex Taq II (TaKaRa Bio Inc., Shiga, Japan) and a LightCycler 96 (Roche Diagnostics, Indianapolis, IN), with the reported primer set (NFWD and NREV2, 142 bp long target in the N gene) (1). Standard curve was generated in duplicate by serial dilutions of the plasmid coding N protein of the Malaysia Strain, pTM-N. The cycling conditions used were 95°C for 30 sec, followed by 45 cycles of 95°C for 5 sec and 60°C for 30 sec, and one cycle of a dissociation step at 95°C for 10 sec, 65°C for 60 sec, and 97°C for 1 sec.

1. Feldman KS, Foord A, Heine HG, et al. Design and evaluation of consensus PCR assays for henipaviruses. *J Virol Methods*. 2009;161(1):52-57. 2.


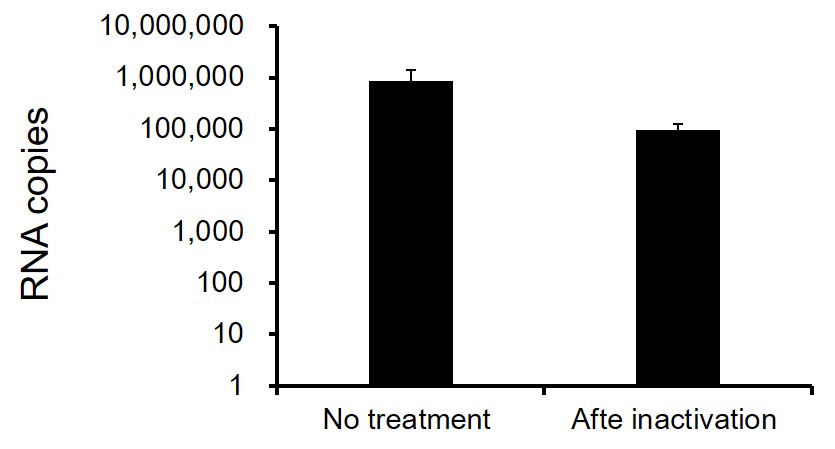


**Supplementary Figure 1.** Quantification of NiV RNAs of the serum sample with spiked NiVs containing 6.0 ×10^5^ TCID_50_ (90% human serum) before or after the inactivation treatment by the protocol shown in Figure 1. The data indicate means ± standard deviations (SD).
